# Elementos para Implementação da Otimização Perioperatória em Cirurgia Cardíaca Baseada no Conceito de “Enhanced Recovery After Surgery”

**DOI:** 10.36660/abc.20240599

**Published:** 2025-04-17

**Authors:** Marcelo Jamus Rodrigues, Andréa de Fátima Cristino Bastos Crespo, Gabrielle Barbosa Borgomoni, Fábio Antônio Serra de Lima, Paola Keese Montanhesi, Luiz Fernando Falcão, Valter Furlan, Omar Asdrúbal Vilca Mejia

**Affiliations:** 1 Hospital Samaritano Paulista São Paulo SP Brasil Hospital Samaritano Paulista, São Paulo, SP – Brasil; 2 Hospital das Clínicas Faculdade de Medicina Universidade de São Paulo São Paulo SP Brasil Serviço de Cirurgia Bariátrica do Hospital das Clínicas da Faculdade de Medicina da Universidade de São Paulo, São Paulo, SP – Brasil; 3 Instituto do Coração do Hospital das Clínicas Faculdade de Medicina Universidade de São Paulo São Paulo SP Brasil Instituto do Coração do Hospital das Clínicas da Faculdade de Medicina da Universidade de São Paulo, São Paulo, SP – Brasil; 4 Hospital Israelita Albert Einstein São Paulo SP Brasil Hospital Israelita Albert Einstein, São Paulo, SP – Brasil; 5 UNIFESP São Paulo SP Brasil Universidade Federal de São Paulo (UNIFESP), São Paulo, SP – Brasil

**Keywords:** Recuperação Pós-Cirúrgica Melhorada, Procedimentos Cirúrgicos Cardiovasculares, Protocolos Clínicos

## Introdução

A otimização da recuperação após cirurgia, proposta pelo conceito de *Enhanced Recovery After Surgery* (ERAS), implica cuidados perioperatórios com o objetivo de minimizar o estresse emocional e fisiológico, acelerando o retorno funcional dos pacientes.^1^

O ERAS emprega *bundles* (pacotes) de intervenções que, em conjunto, reduzem o tempo de internação,^2-4^ o uso de opioides,^4^ as complicações pós-operatórias^2,5^ e os custos hospitalares.^1,6^

Os protocolos que se fundamentam no conceito ERAS têm angariado ampla aceitação em diversas especialidades cirúrgicas^1^ e em instituições de saúde globalmente (www.erassociety.org). No que diz respeito à cirurgia cardíaca, sua implementação é relativamente nova e permanece subutilizada,^7^ o que evidencia peculiaridades dessa especialidade.^8^ Além disso, persistem desafios e dúvidas relacionados à aplicação prática dos protocolos, inclusive quanto à formação das equipes de trabalho, aos métodos para avaliação, ao estímulo à adesão e à necessidade de uma metodologia para o aprimoramento contínuo.

O presente texto apresenta os principais elementos para a implementação de um programa de recuperação fundamentado nos princípios do ERAS no contexto da cirurgia cardíaca.

## Métodos

Trata-se de um estudo de revisão narrativa para o qual se utilizou a base de dados eletrônica MEDLINE como referência. Para a realização do estudo, buscaram-se os termos-chave “*Enhanced Recovery After Surgery*”, “*Cardiac Surgery*”, “*ERAS*”, “*ERAS Cardiac*” e “*implementation*”. Esta pesquisa incluiu artigos publicados nos últimos dez anos, excluindo relatos de caso, comentários, cartas e estudos em outras especialidades. A revisão da literatura se somou à experiência dos autores na implementação de programas de “Otimização da Recuperação”, além de qualidade e segurança na cirurgia cardíaca em três hospitais de referência em Cardiologia localizados no estado de São Paulo, Brasil.^9-11^

### O processo de implementação

#### Diagnóstico situacional

A implementação do ERAS implica o desafio de gerenciamento de mudanças, particularmente na modificação de práticas clínicas e na transformação da cultura organizacional.^12^ Para desenvolver um programa ERAS, é necessário realizar uma análise minuciosa do modelo atual através de duas abordagens principais:^12^

A primeira delas consiste em avaliar os processos vigentes. Os questionamentos a seguir podem nortear essa etapa: os procedimentos estão claramente delineados nos protocolos? Qual é o grau de variação e onde ocorre? A comunicação da equipe é consistente? A infraestrutura é adequada? A educação dos pacientes é eficaz?

A segunda abordagem engloba uma análise dos resultados atuais e a identificação de pontos de baixo desempenho que precisam de melhorias. Cada hospital possui suas particularidades, o que demanda a criação de um programa ERAS adaptado à sua realidade.^13,14^

#### Criação de um grupo coordenador

**Figura 1:**
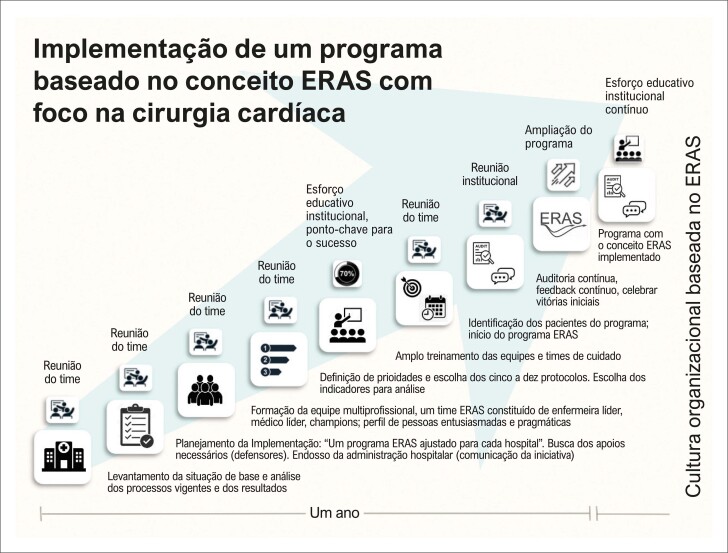
– Fluxograma da implementação de um programa baseado no conceito ERAS com foco na cirurgia cardíaca durante o período de um ano.

É essencial comunicar a importância da mudança e identificar líderes que possam levar a mensagem em cada etapa do cuidado. Montar uma equipe composta de pessoas entusiasmadas, influentes e dinâmicas é fundamental para a implementação do programa, definição de metas e avaliação constante dos protocolos empregados (Figura 1).^12,13^

A equipe deve ser multiprofissional,^1,12-15^ variando conforme os recursos, os interesses e a disponibilidade. A formação de uma equipe de líderes deve ser limitada em número, mas diversa em perspectivas, com reuniões periódicas e comunicação eficaz.^14^

O envolvimento dos médicos é fundamental para o sucesso do programa,^12^ e a nomeação de uma enfermeira coordenadora para orientar o paciente na jornada perioperatória pode gerar um impacto significativo na recuperação.^14^ O endosso da administração hospitalar é essencial para assegurar uma ampla colaboração entre as partes.^12,13^ Compreendemos que uma equipe mínima para a implementação inicial do programa deve contemplar os seguintes profissionais: anestesista, cardiologista, cirurgião cardíaco, intensivista, enfermeiro e fisioterapeuta.

Dado que o ERAS incorpora novos paradigmas, a educação de todos os envolvidos na assistência aos pacientes é essencial.^12,13^ É importante compreender que a otimização de recuperação representa um programa abrangente e um modelo de cuidado, e não somente um protocolo específico.^12^ Um desafio frequente no contexto do ERAS é a variação na assistência em função das fortes preferências médicas pessoais.^13^ Para solucionar esse problema, alguns programas envolvem líderes médicos em suas áreas a fim de criar “*Champions”*, profissionais que promovem a excelência e a inovação no atendimento ao paciente.^14,16^ Fundamentar os protocolos nas melhores evidências disponíveis^17,18^ garante que haja objetividade e foco no paciente, evitando, assim, preferências pessoais excessivas.

A capacitação da equipe é um processo contínuo que combina discussões, reuniões, simpósios, folhetos informativos e material para intranet com os protocolos vigentes, checklists,^12^ literatura de apoio e os contatos dos responsáveis. Durante a fase inicial, a liderança deve estar presente nas unidades de modo a reforçar a importância do ERAS e demonstrar compromisso.^12^

## Definição dos processos

**Figura 2:**
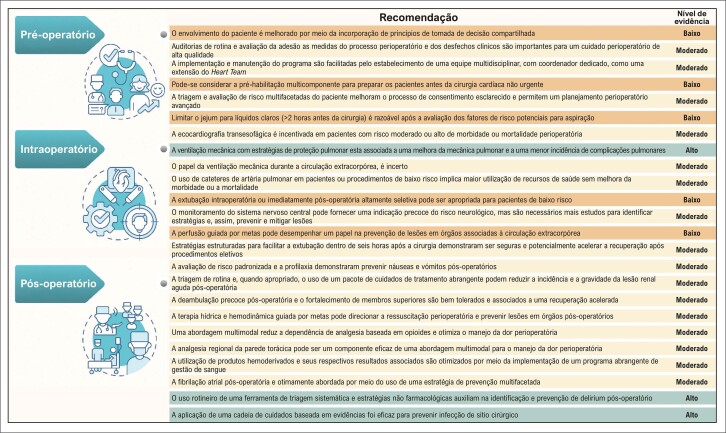
– Recomendações e seus níveis de evidência correspondentes, adaptado de Grant et al.18

Outro passo importante é determinar os processos e os protocolos específicos do ERAS que serão incorporados ao programa de cirurgia cardíaca, que são, de modo geral, estruturados nas três fases do cuidado: pré-operatório, intraoperatório e pós-operatório. Um bom ponto de partida é examinar as recomendações perioperatórias para Cirurgia Cardíaca elaboradas pelas instituições ERAS *Cardiac Society*, ERAS *International Society* e *Society of Thoracic Surgeons* (Figura 2).^17,18^

Embora sejam reconhecidos mais de 25 protocolos perioperatórios, um programa em fase de implementação tem mais sucesso ao adotar de cinco a dez medidas.^12^ A escolha depende da experiência local,^14^ de recursos e de atitudes da equipe. Certas recomendações são fundamentais para um programa ERAS, tais como a educação do paciente,^12,13^ a abreviação do jejum, a analgesia multimodal com redução de opioides e a extubação e a mobilização precoces.^1^ É crucial simplificar os processos e evitar intervenções complexas e de alto custo no início do programa.

## Definição dos pacientes-alvo e educação

Para a eficácia de um programa ERAS, é vital definir o perfil dos pacientes. Uma abordagem habitual é incluir todos os pacientes submetidos a um tipo específico de cirurgia^13^ ou, inicialmente, direcionar o foco para subgrupos de menor risco. Com o tempo, a elegibilidade é expandida de forma a abranger pacientes de maior risco. O envolvimento do paciente no próprio cuidado é indispensável, exigindo educação pré-operatória minuciosa, metas claras, como cessação do tabagismo, orientações dietéticas e atividade física. Materiais informativos também podem ser oferecidos aos pacientes.

## Implementação e auditoria dos processos

**Figura 3:**
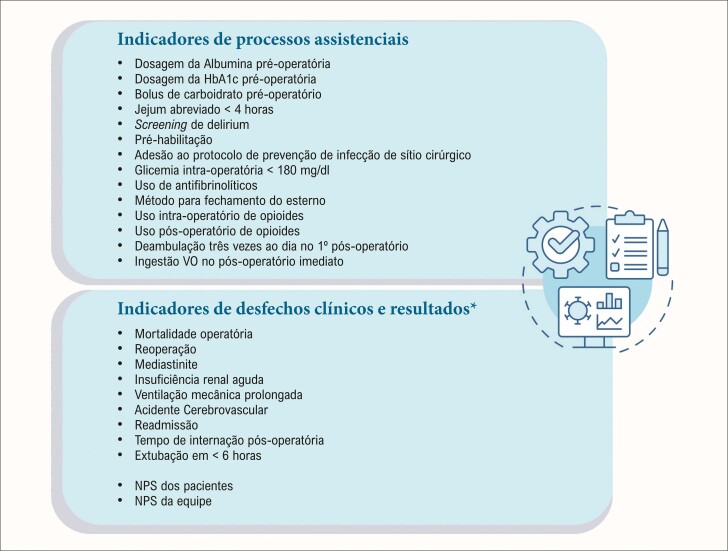
– Lista de indicadores de processos assistenciais, desfechos clínicos e resultados. Com base nas descrições das medidas de desempenho da STS (The Society of Thoracic Surgeons) e na métrica NPS (Net Promoter Score).

A equipe deve determinar quais indicadores do processo assistencial e de desfechos clínicos serão monitorados (figura 3), de forma a quantificar o efeito da implementação do programa. Dois dos elementos mais determinantes e desafiadores da etapa de implementação são a supervisão da adesão às novas diretrizes de cuidados e a avaliação do progresso em direção às metas estabelecidas, uma vez que a conformidade com os protocolos está diretamente associada à melhora dos desfechos clínicos.^3,19^

O programa deve ser reforçado continuamente por meio de atualizações periódicas de desempenho,^12,14^ verificações constantes da literatura,^20^ encorajamento de comparações entre pares e a transparência dos resultados para todas as equipes assistentes e a administração hospitalar. Por fim, celebrar pequenas vitórias^13^ incentivará esforços adicionais.

Embora possa variar, é razoável esperar que o tempo para a efetiva implementação do novo programa de cuidados baseados no ERAS seja de cerca de um ano.^14^

## Conclusão

Implementar um programa de otimização da recuperação após cirurgia cardíaca, fundamentado nos princípios do ERAS, é um processo desafiador que exige atenção ao amplo espectro do cuidado perioperatório e a diversas intervenções. O sucesso do programa requer não apenas habilidades técnicas, mas também competências não técnicas,^12^ como trabalho em equipe, liderança, mudança cultural e gestão das complexidades organizacionais.

Amparados nos princípios do *Enhanced Recovery After Surgery* (ERAS), oferecemos uma estrutura conceitual composta por elementos essenciais e padronizados que promovem cuidados de valor,^1^ orientados por evidências e integrados à cultura local. Essa abordagem destaca a importância da colaboração multidisciplinar e do cuidado centrado no paciente, com monitoramento contínuo e adaptação às necessidades institucionais.^17,18^
