# Supplementary material for: Elementos para Implementação da Otimização Perioperatória em Cirurgia Cardíaca Baseada no Conceito de “Enhanced Recovery After Surgery”
Source: Arq Bras Cardiol. 2025 Apr 17;122(4):e20240599. [Article in Portuguese] doi: 10.36660/abc.20240599 (PMC12107834; doi:10.36660/abc.20240599)
Supplement: Translation

# Translation: Elements for Implementing Perioperative Optimization in Cardiac Surgery Based on Enhanced Recovery after Surgery

**DOI:** 10.36660/abc.20240599i

**Authors:** Marcelo Jamus Rodrigues, Andréa de Fátima Cristino Bastos Crespo, Gabrielle Barbosa Borgomoni, Fábio Antônio Serra de Lima, Paola Keese Montanhesi, Luiz Fernando Falcão, Valter Furlan, Omar Asdrúbal Vilca Mejia

**Affiliations:** 1 Brazil Hospital Samaritano Paulista, São Paulo, SP – Brazil; 2 Brazil Serviço de Cirurgia Bariátrica do Hospital das Clínicas da Faculdade de Medicina da Universidade de São Paulo, São Paulo, SP – Brazil; 3 Brazil Instituto do Coração do Hospital das Clínicas da Faculdade de Medicina da Universidade de São Paulo, São Paulo, SP – Brazil; 4 Brazil Hospital Israelita Albert Einstein, São Paulo, SP – Brazil; 5 Brazil Universidade Federal de São Paulo (UNIFESP), São Paulo, SP – Brazil

**Keywords:** Enhanced Recovery After Surgery, Cardiovascular Surgical Procedures, Clinical Protocols

## Introduction

The optimization of recovery after surgery, as proposed by the Enhanced Recovery After Surgery (ERAS) concept, involves perioperative care aimed at minimizing emotional and physiological stress while accelerating patients’ functional recovery.^1^

ERAS utilizes intervention bundles that, collectively, decrease hospitalization time,^2-4^ opioid use,^4^ postoperative complications,^2,5^ and healthcare expenses.^1,6^

ERAS-based protocols have gained widespread acceptance across multiple surgical specialties^1^ and healthcare institutions worldwide (www.erassociety.org). Their implementation in cardiac surgery is relatively recent and still underused,^7^reflecting the particularities of this specialty.^8^ Additionally, challenges and uncertainties persist regarding the practical application of these protocols, including team training, assessment methods, strategies to encourage adherence, and the need for a methodology for continuous improvement.

This paper outlines the key elements for implementing a recovery program grounded in ERAS principles within cardiac surgery.

## Methods

This is a narrative review study conducted using the MEDLINE electronic database to search for the key terms “Enhanced Recovery After Surgery,” “Cardiac Surgery,” “ERAS,” “ERAS Cardiac,” and “implementation.” This research included articles published in the last decade, except for case reports, commentary articles, letters, and studies in other specialties. The literature review was complemented by the authors’ experience in implementing “Recovery Optimization” programs, as well as quality and safety initiatives in Cardiac Surgery at three leading hospitals for cardiology in the state of São Paulo, Brazil.^9-11^

### Implementation process

#### Situational diagnosis

Implementing ERAS involves the challenge of change management, particularly in altering clinical practices and transforming organizational culture.^12^ Developing an ERAS-based program requires a detailed analysis of the current model using two primary approaches:^12^

The first approach consists of assessing the existing processes. The following questions can help guide this phase: Are the procedures clearly defined in the protocols? What is the degree of variation, and where does it occur? Is the team’s communication consistent? Is the infrastructure adequate? Is patient education effective?

The second approach encompasses analyzing the current outcomes and identifying low-performance areas that need improvement. Each hospital has unique characteristics that require the development of an ERAS-based program tailored to its specific context.^13,14^

#### Establishing a coordinating group

It is essential to communicate the importance of the change and identify leaders to convey the message at every care stage. Building a team of motivated, influential, and dynamic individuals is key to implementing the program, setting goals, and regularly reassessing the protocols in use (Figure 1).^12,13^

**Figure 1:** – Flowchart for the implementation of an ERAS-based program in cardiac surgery over one year.

The team must be multidisciplinary,^1,12-15^ varying according to resources, interests, and availability. The formation of a leadership team should be limited in number but diverse in perspectives, with regular meetings and efficient communication.^14^

The involvement of physicians is essential to the success of the program,^12^ and appointing a coordinator nurse to guide the patient through the perioperative process can have a significant impact on recovery.^14^ Hospital administration endorsement is crucial to ensure broad collaboration.^12,13^ A minimum team for the initial implementation of the program should consist of: anesthesiologist, cardiologist, cardiac surgeon, intensivist, nurse, and physiotherapist.

Since ERAS introduces new paradigms, training all those involved in patient care is essential.^12,13^ It is important to understand that recovery optimization represents a comprehensive program, a care model, and not just a specific protocol.^12^ A common challenge in the ERAS context is the variation in care due to strong personal medical preferences.^13^ To address this, some programs engage medical leaders in their specialties to create “Champions,” professionals who advocate for excellence and innovation in patient care.^14,16^ Grounding protocols in the best available evidence^17,18^ ensures objectivity and focuses care on the patient, thus minimizing excessive personal preferences.

Team training is a continuous process that combines discussions, meetings, symposiums, informational leaflets, and intranet materials with existing protocols, checklists,^12^ supporting literature, and contact information for responsible parties. Leadership should be present in the units in the early stages to reinforce the importance of ERAS and to show commitment.^12^

## Defining Processes

Another key step is to define the specific ERAS processes and protocols to be integrated into the cardiac surgery program, typically organized into three phases of care: preoperative, intraoperative, and postoperative. A solid starting point is to review the perioperative guidelines for Cardiac Surgery developed by the ERAS Cardiac Society, ERAS International Society, and the Society of Thoracic Surgeons (Figure 2).^17,18^

**Figure 2:** – Guidelines and their levels of evidence, adapted from Grant et al.18

Although more than 25 perioperative protocols are recognized, a program in the implementation phase is more successful when adopting five to ten measures.^12^ The selection depends on local experience,^14^ available resources, and team’s mindsets. Certain guidelines are central to an ERAS-based program, such as patient education,^12,13^ shortened fasting, multimodal analgesia with opioid reduction, and early extubation and mobilization.^1^ It is crucial to keep processes simple and avoid complex, high-cost interventions at the beginning of the program.

## Target patient selection and education

Defining the patient profile is a key factor in the effectiveness of an ERAS-based program. A common approach is to include all patients undergoing a specific type of surgery^13^ or, initially, focus on lower-risk subgroups. Over time, eligibility expands to include higher-risk patients. Patients’ participation in their own care is crucial and requires detailed preoperative education, clear goals such as smoking cessation, dietary guidance, and physical activity recommendations. Supplementary information materials can also be provided to patients.

## Implementation and audit of processes

The team must determine which care process and clinical outcome indicators will be monitored (Figure 3) to quantify the program’s impact. One of the most critical and challenging aspects of implementation is overseeing adherence to new care guidelines and assessing progress toward established goals, as compliance with protocols is directly correlated with better clinical outcomes.^3,19^

**Figure 3:** – List of care process, clinical outcome and endpoint indicators. Based on the performance measures from the Society of Thoracic Surgeons (STS) and NPS (Net Promoter Score).

The program should be continuously reinforced through regular performance updates,^12,14^ periodic literature reviews,^20^ encouragement of peer comparisons, and transparency of results for all care teams, and hospital administration. Finally, celebrating small victories^13^ will encourage further efforts.

Although it may vary, it is reasonable to expect that the effective implementation of a new ERAS-based care program will take about one year.^14^

## Conclusion

The implementation of a recovery optimization program following cardiac surgery, grounded in the principles of ERAS, is a complex process that demands focus on the entire perioperative care spectrum and multiple interventions. Success requires not only technical skills but also non-technical competencies,^12^ such as teamwork, leadership, cultural change, and management of organizational complexities.

Rooted in the principles of Enhanced Recovery After Surgery (ERAS), we present a conceptual framework comprising essential, standardized elements that foster value-based care,^1^ supported by evidence and integrated into the local culture. This approach highlights the importance of multidisciplinary collaboration and patient-centered care, with continuous monitoring and adaptation to institutional needs.^17,18^
